# Supplementary material for: A Perturbed Asymmetrical Y-TypeSheathless Chip for Particle Control Based on Adjustable Tilted-Angle Traveling Surface Acoustic Waves (ataTSAWs)
Source: Biosensors (Basel). 2022 Aug 7;12(8):611. doi: 10.3390/bios12080611 (PMC9406206; doi:10.3390/bios12080611)
Supplement: Supplementary file 1 [file biosensors-12-00611-s001.zip › Figure S1.pdf]

We set up locating points on the microchannel and substrate to realize the combination of different angles. Among them, there is a pair of locating points in the microchannel (Figure S1a). The number of the substrate locating points was consistent with the number of angle species. In this paper, there are 5 pairs, corresponding to 5°, 15°, 25°, 35° and 45° respectively (Figure S1b). In the experiment, with the help of a magnifying glass and tweezers, the microchannel was moved to align it with the substrate at an angle. Figure S1c-g show the schematic diagram of the combination of the microchannel and substrate at five different angles.

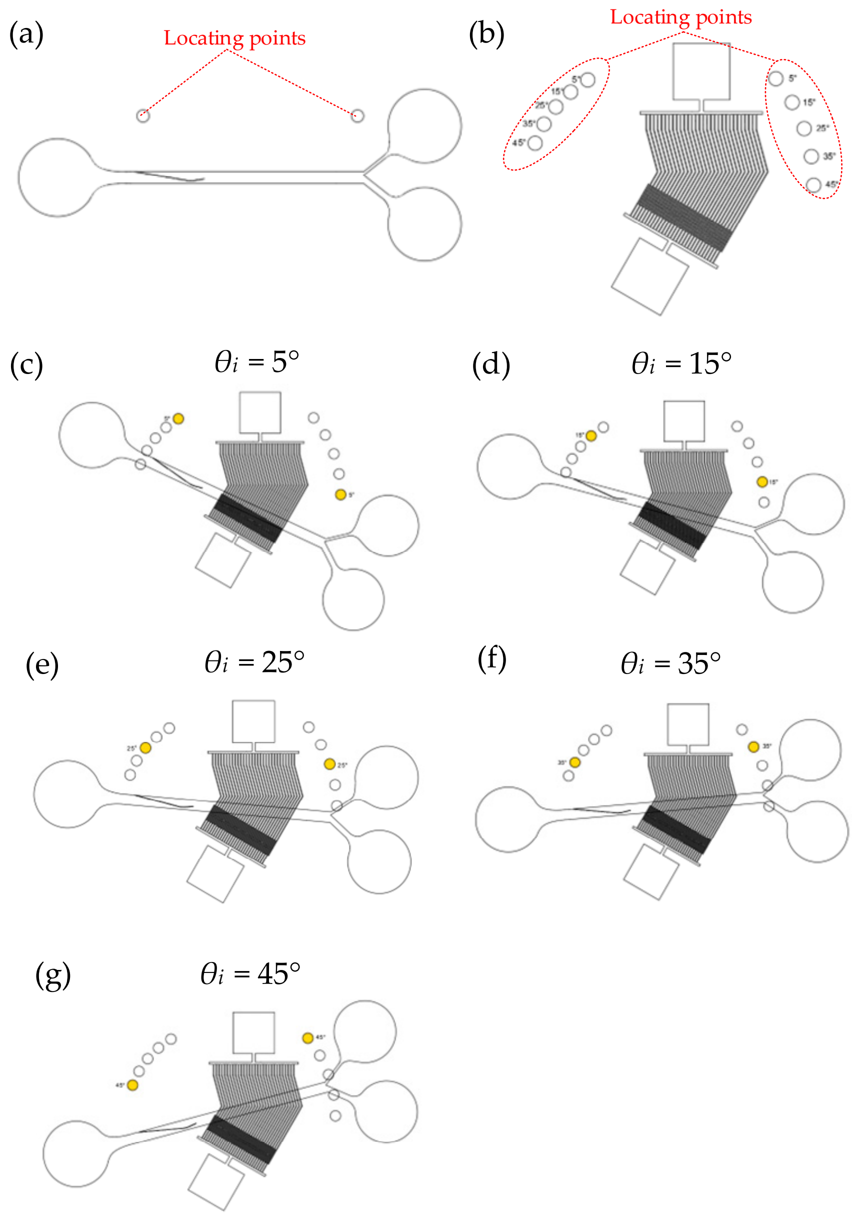

Figure S1. Schematic diagram of locating points on the microchannel and the substrate.
