# Supplementary material for: A Perturbed Asymmetrical Y-TypeSheathless Chip for Particle Control Based on Adjustable Tilted-Angle Traveling Surface Acoustic Waves (ataTSAWs)
Source: Biosensors (Basel). 2022 Aug 7;12(8):611. doi: 10.3390/bios12080611 (PMC9406206; doi:10.3390/bios12080611)
Supplement: Supplementary file 1 [file biosensors-12-00611-s001.zip › Figure S2,Table S1.pdf]

With the help of COMSOL software, we carry on the simulation analysis to the designed surface acoustic wave (SAW) device. The structure parameters are as follows: the base material is 128°Y-X tangential lithium niobate, the electrode fork finger width a is 29 μm, the finger spacing p is 58 μm, and the electrode thickness is 0.1 μm. After analyzing the characteristic frequency (f_c) of 30 MHz-40 MHz for the established model, it is concluded that the designed device can work in traveling-wave mode when f_c = 33.529 MHz (as shown in Figure S2). Meanwhile, the wave velocities (v_s) of the substrate surface at different frequencies were obtained (as shown in Table S1). It can be concluded that when f_c =33.529 MHz, the wave velocity of the SAW device is 3889.4 m/s. The simulation analysis of the characteristic frequency provides help for our subsequent experimental test (an excitation near a certain frequency is applied to the SAW device to make it work in traveling-wave mode).

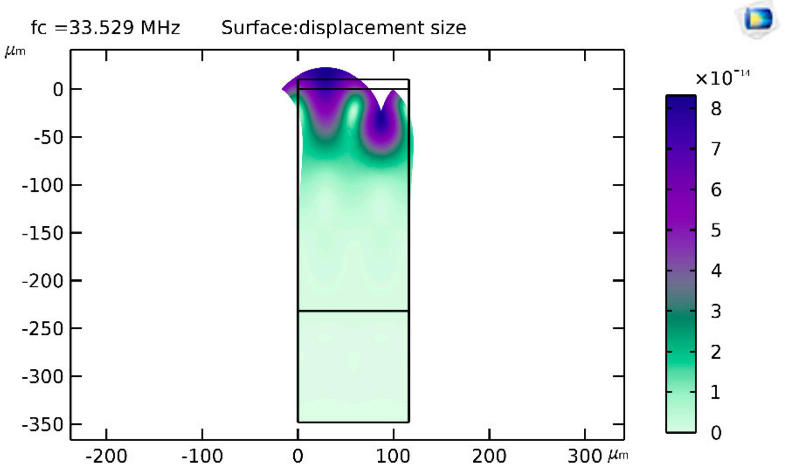

Figure S2. Characteristic frequency simulation diagram of SAW device. When f_c =33.529MHz, SAW device can work in traveling-wave mode.

Table S1. Wave velocity of substrate surface at different frequencies

f_c (MHz)	v_s (m/s)
31.749+9.2756i	3682.8+1076.0i
32.236+10.736i	3739.4+1245.4i
33.529	3889.4
33.974	3940.9
38.834+8.2929i	4504.7+961.97i
